# Liquid demixing of intrinsically disordered proteins is seeded by poly(ADP-ribose)

**DOI:** 10.1038/ncomms9088

**Published:** 2015-08-19

**Authors:** Matthias Altmeyer, Kai J. Neelsen, Federico Teloni, Irina Pozdnyakova, Stefania Pellegrino, Merete Grøfte, Maj-Britt Druedahl Rask, Werner Streicher, Stephanie Jungmichel, Michael Lund Nielsen, Jiri Lukas

**Affiliations:** 1 Novo Nordisk Foundation Center for Protein Research, Faculty of Health and Medical Sciences, University of Copenhagen, Blegdamsvej 3b, Copenhagen DK-2200, Denmark; 2 Institute of Veterinary Biochemistry and Molecular Biology, University of Zurich, Winterthurerstrasse 190, Zurich CH-8057, Switzerland

## Abstract

Intrinsically disordered proteins can phase separate from the soluble intracellular space, and tend to aggregate under pathological conditions. The physiological functions and molecular triggers of liquid demixing by phase separation are not well understood. Here we show *in vitro* and *in vivo* that the nucleic acid-mimicking biopolymer poly(ADP-ribose) (PAR) nucleates intracellular liquid demixing. PAR levels are markedly induced at sites of DNA damage, and we provide evidence that PAR-seeded liquid demixing results in rapid, yet transient and fully reversible assembly of various intrinsically disordered proteins at DNA break sites. Demixing, which relies on electrostatic interactions between positively charged RGG repeats and negatively charged PAR, is amplified by aggregation-prone prion-like domains, and orchestrates the earliest cellular responses to DNA breakage. We propose that PAR-seeded liquid demixing is a general mechanism to dynamically reorganize the soluble nuclear space with implications for pathological protein aggregation caused by derailed phase separation.

## Abstract (web summary)

Intrinsically disordered proteins can phase separate from the soluble intracellular space. Here the authors show that the nucleic acid-mimicking biopolymer poly(ADP-ribose) (PAR) nucleates intracellular liquid demixing and orchestrates the earliest cellular responses to DNA breakage.

## Graphical abstract

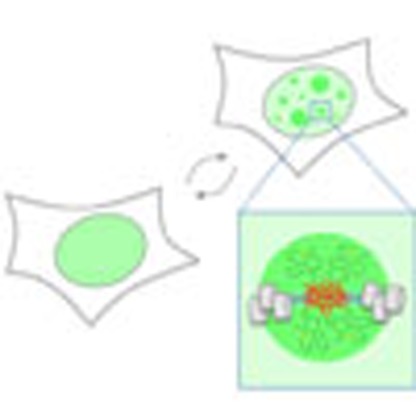

The discovery of intrinsically disordered proteins (IDPs) has challenged the view that protein function depends on a defined three-dimensional structure, and their recently unveiled involvement in diseases marked by pathological protein aggregation has raised significant interest in their biochemical properties and cellular functions^1^,^2^,^3^. Many IDPs contain low complexity domains (LCDs), unstructured protein sequences composed of repetitive sequence elements that tend to phase separate into liquid droplets, very much like oil undergoes liquid demixing (also referred to as liquid–liquid demixing) in aqueous solutions^4^,^5^,^6^,^7^. In cells, such liquid demixing events have the potential to dynamically organize the soluble intracellular space into spatially confined compartments^8^, but what initiates such phase separations has remained largely elusive. The formation of a liquid droplet by phase separation can be triggered by nucleation, occurring either spontaneously via random collisions of molecules or at specific pre-existing subcellular sites^8^. In the case of LCD-containing IDPs, certain RNA species can initiate their assembly into higher-order structures^9^. However, other nucleation or seeding events might exist. An exciting possibility is that nucleation may not only occur on pre-existing sites but also via the *de novo* generation of molecular seeds. Such a mechanism would provide cells with the opportunity to respond to internal and external cues, for instance under conditions of cell stress, by context- and site-specific compartmentalization through liquid demixing and without the need for physical membrane barriers.

Here we show both *in vitro* and in the physiological context of damaged chromatin in human cells that the formation of PAR represents an inducible molecular seed for the assembly of IDPs, and thereby initiates liquid demixing to achieve dynamic intracellular compartmentalization. Two types of LCDs participate in this process: positively charged arginine–glycine–glycine (RGG) repeats, which act as a PAR sensor, and prion-like protein domains, which amplify the PAR-seeded liquid demixing. The assembly of IDPs at DNA break sites is short-lived and countered by subsequent protein exclusion from damaged areas. We propose that dynamic liquid demixing of proteins with LCDs is a general function of localized PAR formation through activation of PARP enzymes, and that such phase separations orchestrate DNA repair reactions and promote genome stability maintenance.

## Results

### PAR triggers IDP assembly at sites of DNA damage

**Figure 1:**
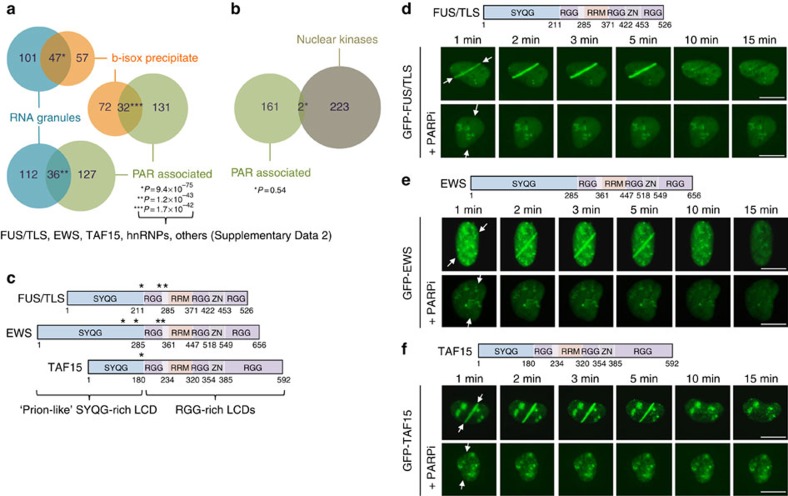
Intrinsically disordered proteins accumulate at sites of DNA damage in a PAR-dependent manner. (**a**) Overlap of proteins associated with RNA granules (blue), b-isox precipitates and *in vitro* generated hydrogels (orange), and PAR (green). (**b**) Overlap of proteins associated with PAR (green) and a control group of 225 nuclear kinases (brown). (**a**,**b**) Asterisks indicate right-tailed *P* values (Fisher's exact test). (**c**) Protein domain organization of the LCD-containing FET proteins FUS, EWS and TAF15, each harbouring prion-like SYQG-rich amino termini and carboxyl termini rich in RGG repeats. Asterisks indicate regions of oncogenic translocations. (**d**) Recruitment kinetics of GFP–FUS to sites of laser microirradiation in the absence or presence of PARP inhibitor olaparib (10 μM). Time-lapse movie stills from the first 15 min after irradiation are shown. White arrows indicate the orientation of the laser line. See also Supplementary Movie 1. (**e**) Recruitment kinetics of GFP–EWS to sites of laser microirradiation. (**f**) Recruitment kinetics of GFP–TAF15 to sites of laser microirradiation. Scale bars, 10 μm.

On the basis of multivalent anionic nature of PAR, its nucleic acid-like properties, and the fact that PAR formation is tightly regulated with PAR levels rising mainly under conditions of cell stress and in particular in response to DNA damage (Supplementary Fig. 1a), we devised the hypothesis that PAR formation might represent a nucleation event to initiate site-specific liquid demixing of LCD-containing proteins. In support of this presumption, we had previously identified an LCD in the protein SAFB1 (scaffold attachment factor B1) as a sensor of PAR formation that was required and sufficient for the recruitment to sites of DNA damage^10^. Protein knowledge database inquiries using the PrePPI database^11^ indicated potential interactions between SAFB1 and various other LCD-containing RNA-binding proteins (Supplementary Data 1). Moreover, we noticed a significant overlap between the proteins capable of undergoing liquid demixing^5^ and proteins previously identified by proteomics approaches to be associated with PAR^12^ (Fig. 1a). In contrast, no significant overlap was found between PAR-associated proteins and a control group containing 225 nuclear protein kinases (Fig. 1b; Supplementary Fig. 1b). Among the PAR-associated LCD-containing proteins were the IDPs FUS/TLS (fused in sarcoma/translocated in sarcoma), EWS (ewing sarcoma), TAF15 (TATA box-binding protein-associated factor 68 kDa) (collectively abbreviated as FET proteins, also referred to as the TET family of proteins) and a number of heterogenous nuclear ribonucleoproteins (hnRNPs; Supplementary Data 2). Especially the three FET proteins caught our attention for the following reasons: first, various point mutations within the FET genes have been associated with pathological protein aggregation in neurodegenerative diseases, in particular with amyotrophic lateral sclerosis (ALS), and frontotemporal lobar degeneration (FTLD)^2^. One way how such mutations can contribute to proteinopathies is by increasing the propensities for intracellular aggregation, as recently demonstrated for FUS^13^. Second, the FET proteins frequently show gene translocation in human cancers, and emerging evidence suggests their physical interactions with DNA lesions and thus involvement in genome integrity maintenance^14^,^15^,^16^,^17^,^18^. Third, due to their high degree of intrinsic structural disorder, these proteins are prototype IDPs, each harbouring a prion-like SYQG-rich amino-terminal LCD and an extended RGG-rich carboxyl-terminal LCD-containing 18–22 RGG repeats (Fig. 1c). We thus set out to test the hypothesis that PAR might seed phase separation and liquid demixing using the three FET proteins as prototype IDPs and the cellular response to DNA damage as model system, exploiting the fact that PAR levels are highly induced at sites of DNA breaks^19^.

To locally induce DNA damage, we employed laser microirradiation protocols developed earlier in our laboratory^20^, which allowed us to monitor the earliest cellular responses to DNA strand breaks in real time and with hitherto unsurpassed temporal resolution. In line with recent work^15^,^16^,^17^, we observed rapid and transient accumulation of green fluorescent protein (GFP)–FUS, GFP–EWS and GFP–TAF15 at sites of DNA damage (Fig. 1d–f, top panels; Supplementary Movie 1). However, and consistent with our hypothesis, we noticed that the accumulation of all the three proteins occurred with similarly fast kinetics and in a strictly PAR-dependent manner (Fig. 1d–f, bottom panels), suggesting that PAR is the common upstream seeding event that initiates the assembly of LCD-containing proteins. We verified that the endogenous protein levels were sufficient to allow FET protein accumulation at sites of DNA damage and that the accumulation of the endogenous proteins followed very similar kinetics as the GFP fusions (Supplementary Fig. 1c), and also coincided with local PAR formation, following the kinetics of its early appearance and subsequent disappearance (Supplementary Fig. 1d–f). Moreover, the PAR-dependent accumulation was not restricted to laser-inflicted DNA damage, because transient and PAR-dependent accumulation of the FET proteins on chromatin was also observed following treatment with hydrogen peroxide, a well established activator of PARP enzymes, which indeed resulted in rapid, cell cycle phase-independent PAR formation that coincided with FET protein accumulation as revealed by quantitative automated microscopy (Supplementary Fig. 2a–d). Together, these data provide evidence that PAR formation is a general trigger for the assembly of prototype LCD-containing proteins. They also suggest that LCD-containing proteins might cooperate with PAR signalling to enhance genome integrity maintenance. We thus reasoned that loss of the FET proteins should sensitize cells to conditions when PAR formation is impaired, but would be epistatic under conditions when PAR formation is completely blocked. Colony formation assays indeed showed sensitization by FET protein knockdown to low concentrations of the PARP inhibitor olaparib, whereas no further decrease in clonogenic survival was observed at high concentrations of the inhibitor (Supplementary Fig. 3). Thus, the FET proteins emerge as effectors of PAR signalling. We therefore set out to investigate the mechanism of their PAR-dependent assembly and, given their high intrinsic propensity to phase separate^5^, whether this process may involve dynamic liquid demixing.

### RGG modules are sensors of PAR formation

**Figure 2:**
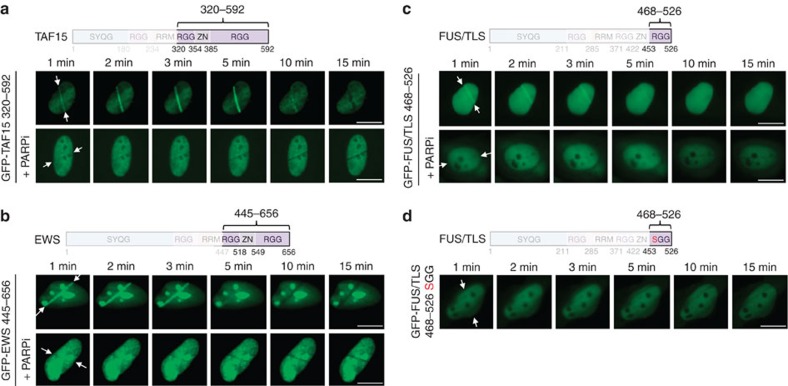
The RGG modules in LCD-containing proteins function as sensors of PAR formation. (**a**) Recruitment kinetics of GFP–TAF15 320–592 (20 RGGs) to sites of laser microirradiation in the absence or presence of PARP inhibitor olaparib (10 μM). Time-lapse movie stills from the first 15 min after irradiation are shown. White arrows indicate the orientation of the laser line. (**b**) Recruitment kinetics of GFP–EWS 445–656 (16 RGGs) to sites of laser microirradiation. (**c**) Recruitment kinetics of GFP–FUS 468–526 (8 RGGs) to sites of laser microirradiation. (**d**) GFP–FUS 468–526 in which RG/RGGs were altered to SG/SGG was expressed; cells were laser microirradiated and imaged as in **a**–**c**. Scale bars, 10 μm.

Since PAR induction is associated with the generation of a high local density of negative charges, we first tested whether the positively charged RGG repeats present in the FET proteins and frequently mutated in ALS/FTLD could be involved in sensing PAR formation. We constructed mutants containing different RGG repeat numbers and tested their accumulation at sites of laser microirradiation. A GFP–TAF15 construct containing 20 out of the original 22 RGG repeats, but lacking the amino-terminal half of the protein, showed pronounced recruitment to the sites of DNA damage in a PAR-dependent manner (Fig. 2a). A GFP–EWS construct containing 16 out of the original 22 RGG repeats also accumulated in a PAR-dependent manner, yet to a slightly reduced extent (Fig. 2b). The shortest GFP–FUS mutant, containing 8 RGG repeats, recruited only weakly, yet measurably and again in a PAR-dependent manner (Fig. 2c). Thus, LCDs rich in RGG repeats appear to sense local PAR accumulation in a repeat number-dependent manner. To test whether the positive charge provided by the arginine residue is required for PAR-dependent assembly, we mutated all eight arginines present in the short GFP–FUS fragment with serines. In contrast to the wild-type construct, the SGG mutant failed to accumulate at sites of laser microirradiation (Fig. 2d). Thus, the positive charges in RGG repeats are indeed necessary for electrostatic PAR interaction.

### Prion-like domains phase separate by liquid demixing

**Figure 3:**
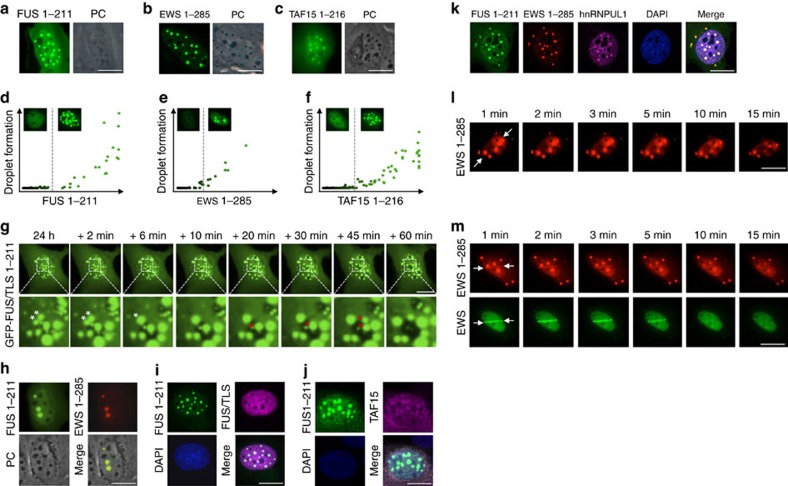
Prion-like domains of LCD-containing proteins phase separate to form homotypic and heterotypic droplets by liquid demixing. (**a**) GFP–FUS 1–211 was expressed for 24 h in U-2-OS cells and spontaneous intracellular droplet formation was detected by fluorescence and phase-contrast (PC) microscopy. (**b**) GFP–EWS 1–285 was expressed and analysed as in **a**. (**c**) GFP–TAF15 1–216 was expressed and analysed as in **a**. (**d**) Sub-nuclear formation of GFP–FUS 1–211 droplets, as scored and quantified by software-assisted image analysis using the Olympus ScanR system, is depicted as a function of GFP–FUS 1–211 expression to reveal a sharp transition towards droplet formation after reaching intra-nuclear concentrations sufficient to trigger spontaneous liquid demixing. (**e**) Sub-nuclear formation of GFP–EWS 1–285 was scored and analysed as in **d**. (**f**) Sub-nuclear formation of GFP–TAF15 1–216 was scored and analysed as in **d**. (**g**) GFP–FUS 1–211 was expressed as in **a** and monitored live by time-lapse imaging over a period of 1 h in 2-min intervals. Movie stills and magnifications of intracellular liquid droplets are provided. White asterisks mark a fusion event of two droplets, while red asterisks mark a fission event. See also Supplementary Movie 2. (**h**) GFP–FUS 1–211 and Tomato-EWS 1–285 were co-expressed for 24 h in U-2-OS cells and spontaneous formation of heterotypical droplets was detected by fluorescence and phase-contrast (PC) microscopy. (**i**) GFP–FUS 1–211 was expressed as in **a**, cells were fixed and stained for endogenous FUS using an antibody against the carboxyl terminus of the protein. (**j**) GFP–FUS 1–211 was expressed as in **a**, cells were fixed and stained for endogenous TAF15. (**k**) GFP–FUS 1–211 and Tomato-EWS 1–285 were co-expressed as in **h**, cells were fixed and stained for endogenous hnRNPUL1. (**l**) Tm-EWS 1–285 was expressed for 24 h, cells were laser microirradiated and imaged as in Fig. 2a–d. Time-lapse movie stills from the first 15 min after irradiation are shown. White arrows indicate the orientation of the laser line. (**m**) Tm-EWS 1–285 and GFP–EWS were co-expressed for 24 h, cells were laser microirradiated and imaged as in Fig. 2a–d. Time-lapse movie stills from the first 15 min after irradiation are shown. Scale bars, 10 μm.

The prion-like amino-terminal SYQG-rich domains are intrinsically aggregation-prone sequences that participate in the formation of higher-order assemblies of the FET proteins^21^. We noticed that the isolated amino termini derived from the three FET proteins spontaneously assembled into remarkably spherical structures when expressed in human cells (Fig. 3a–c; Supplementary Fig. 4a). We considered that such spontaneous assemblies resulted from liquid demixing of intrinsically aggregation-prone sequences when intracellular concentrations exceed threshold levels. Indeed, quantitative imaging provided evidence that for all the three FET proteins threshold levels determined the formation of spherical intracellular droplets (Fig. 3d–f). Further, consistent with the idea of liquid droplet formation, time-lapse imaging revealed that the protein assemblies were mobile, underwent fusion and fission events, and grew in size as expression levels increased (Fig. 3g; Supplementary Movie 2). All these features observed for the FET prion-like domains (spherical structure likely due to minimized surface tension, sharp transition towards droplet formation as protein concentrations increase, intracellular mobility, fusion and fission events) are hallmarks of liquid droplets generated by liquid demixing^22^. Strikingly, and fully consistent with compartmentalization by phase separation, we observed that co-expressed prion-like domains of different FET proteins were exclusively found to stably cohabit the same large liquid droplets, indicating that liquid demixing involves homotypic as well as heterotypic interactions among FET proteins, and that heterotypic interactions can contribute to the demixing process (Fig. 3h; Supplementary Fig. 4b).

By definition, liquid demixing generates membrane-less compartments within the subcellular space in which certain components are enriched while others are excluded. A prominent example is the nucleolus, which is highly enriched in ribosomal RNA and ribosomal RNA-binding proteins, but non-permissive for other nuclear components, including certain branches of the DNA damage response. We noticed that the liquid droplets generated by the prion-like domains of the FET proteins resulted in a clearly visible alteration of light diffraction, very comparable to the light diffraction patterns associated with the nucleoli (Fig. 3a–c,h). Intrigued by this finding, we aimed to test whether FET protein assemblies, just like other membrane-less compartments such as the nucleoli, might function as molecular sieve to include some and exclude other cellular components. We thus interrogated endogenous protein associations within FET liquid droplets by indirect immunofluorescence and found that droplets containing the ectopically expressed prion-like amino terminus of FUS also attracted endogenous FUS, as detected by an antibody against its carboxyl terminus (Fig. 3i). Strikingly, it could even locally enrich endogenous TAF15 (Fig. 3j). Moreover, not only the endogenous FET proteins self-assembled *in vivo* into heterotypic liquid droplets but also hnRNPUL1, another LCD-containing genome caretaker^23^,^24^, was present in these spherical structures (Fig. 3k; Supplementary Fig. 4c). Together, these data reveal that the prion-like domains of the FET proteins can self-assemble in a homotypic and heterotypic manner, and that such assemblies locally concentrate additional LCD-containing proteins to further drive phase separation.

In stark contrast to the specific enrichment of LCD-containing proteins in liquid droplets, several globular (that is, fully folded) canonical DDR factors including Ku70, NBS1, MDC1 and 53BP1 were partly excluded from these compartments, as they were from the nucleoli (Supplementary Fig. 4d–g), indicating that LCD protein-mediated phase transitions can indeed function to transiently trap some and exclude other nuclear proteins.

Given the homotypic and heterotypic assembly of the FET proteins into liquid droplets, we next tested whether the prion-like domains could also assemble at sites of DNA damage where PAR levels transiently spike. Under normal conditions, the isolated prion-like domains assembled into sub-nuclear droplets as observed before, but we did not detect any measurable recruitment to sites of laser microirradiation (Fig. 3l). However, when we co-expressed full-length EWS together with its prion-like domain, we observed transient accumulation of both the proteins at DNA damage sites with very comparable kinetics (Fig. 3m). These data substantiate the notion that the FET proteins can form homotypic and heterotypic assemblies, which are at least in part mediated by their prion-like domains, and further indicate that the prion-like domains participate in FET protein assembly at sites of DNA damage. Indeed, and consistent with previous findings^17^, fusing the prion-like domain of FUS (amino acids 1–211) to its carboxyl-terminal RGG repeats (amino acids 468–526) led to more pronounced accumulation at laser-irradiated sites as compared with the RGG-rich carboxyl-terminus alone (Supplementary Fig. 4h). Collectively, these results suggest that the PAR-sensing RGG repeats and the aggregation-prone prion-like domains functionally cooperate to build-up higher-order assemblies of LCD-containing proteins at the sites of DNA damage.

### PAR seeds liquid demixing at sites of DNA damage

**Figure 4:**
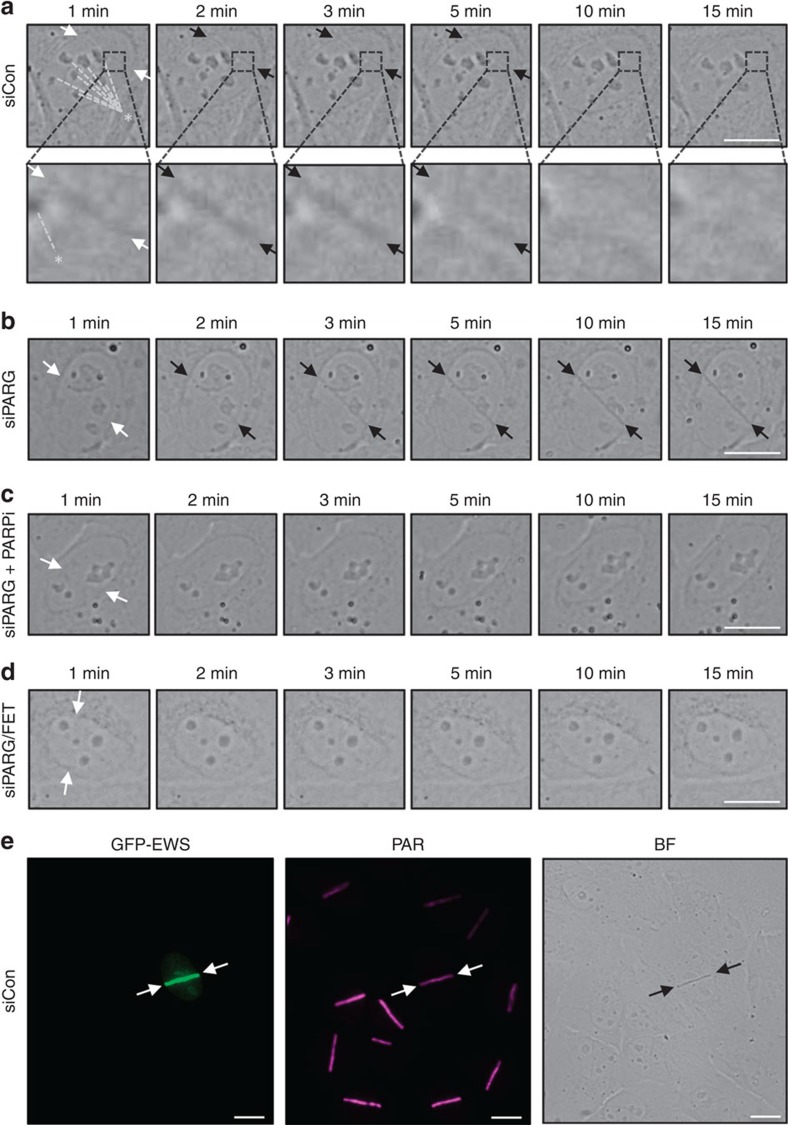
PAR-dependent accumulation of LCD-containing proteins seeds liquid demixing at sites of DNA damage. (**a**) Bright-field images depicting the transient generation of distinct light-diffracting stripes at sites of laser microirradiation under conditions of increased laser energy. White arrows indicate the orientation of the laser line, black arrows point to light-diffracting stripes. Asterisk and dashed lines point to light-diffracting nucleoli. See Supplementary Materials for details. (**b**) Bright-field images depicting the enhanced and prolonged generation of distinct light-diffracting stripes at sites of laser microirradiation in PARG-depleted cells. See also Supplementary Movie 3. (**c**) Bright-field images of laser microirradiated PARG-depleted cells in the presence of PARP inhibitor. (**d**) Bright-field images of laser microirradiated siPARG/FET cells. (**e**) Transient ectopic expression of GFP–EWS enhances the generation of light-diffracting stripes in otherwise naive U-2-OS cells. Following laser microirradiation, cells were fixed and stained for PAR. White arrows in **b**–**e** indicate the orientation of the laser line, black arrows point to light-diffracting stripes. Scale bars, 10 μm.

The above findings are consistent with the idea that spontaneous assemblies of isolated prion-like domains and the DNA damage-associated assemblies of full-length LCD-containing proteins share similar physicochemical properties. We thus suspected that, just like the formation of liquid droplets through prion-like domains, the PAR-dependent assembly of IDPs at sites of DNA damage might change the local nuclear environment to a degree that would be detectable as physical change. To address this point, we employed time-lapse bright-field and phase-contrast live-cell microscopy after microirradiation to monitor the changes in light diffraction associated with the immediate response to DNA damage. To maximize the chance of observing PAR-induced physical changes in living cells, we first applied a moderately higher laser power to increase the local density of DNA breaks. Under these conditions, we consistently observed the transient formation of distinct light-diffracting dark stripes exactly at the microirradiated regions (Fig. 4a), which in terms of light diffraction pattern closely resembled the one described above in the nucleoli and in the FET prion-derived liquid droplets. Since the kinetics of the change in light diffraction mirrored PAR formation and the rapid PAR-dependent protein assemblies at sites of DNA damage (Fig. 1; Supplementary Fig. 1), we reasoned that they could be mechanistically linked to PAR-seeded events, and that an increase in PAR stability might therefore enhance this cellular response. Indeed, when we depleted the PAR-degrading enzyme PAR glycohydrolase (PARG) by short interfering RNA (siRNA), the local increase in light diffraction was more pronounced, lasted considerably longer, and was detectable even after reducing the laser intensity to inflict DNA damage (Fig. 4b; Supplementary Movie 3). The observed change in light diffraction was visible by both bright-field and phase-contrast microscopy, developed over time and was fully reversible (Supplementary Fig. 5a,b), did not occur at microirradiated areas outside the cell nucleus (Supplementary Fig. 5c), and could even be observed under conditions when much smaller areas of the nucleus were microirradiated and thus less DNA damage was induced (Supplementary Fig. 5d), together suggesting that it represents a bona fide physical consequence of the molecular events triggered by DNA damage. Moreover, chemical inhibition of PAR formation by PARP inhibitors completely abolished the transient changes in light diffraction (Fig. 4c) without having an obvious effect on the amount of DNA damage being induced as revealed by clearly visible γH2AX formation along the irradiated laser track (Supplementary Fig. 5e,f). Also, gradual depletion of PARP1/ARTD1 (Supplementary Fig. 5g), the enzyme mainly responsible for genotoxic stress-induced PAR formation, reduced both local PAR induction (Supplementary Fig. 5h) and the accompanying alteration in light diffraction in a dose-dependent manner (Supplementary Fig. 5i), reinforcing that PARylation controls this cellular response.

**Figure 5:**
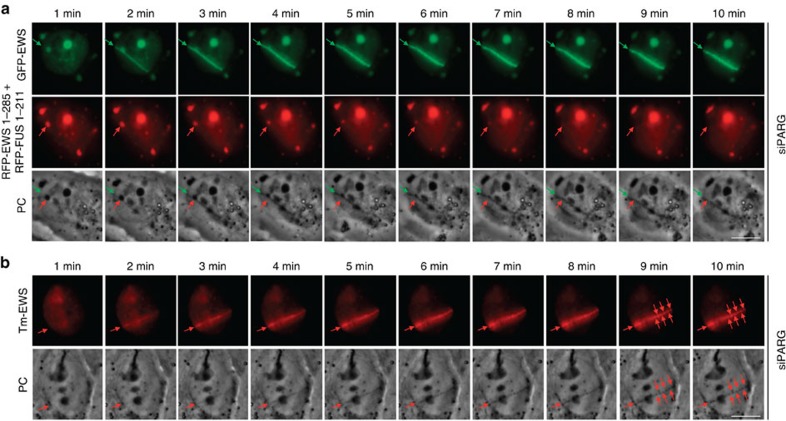
Liquid demixing of LCD-containing proteins is dynamic and phase separated compartments can exchange its constituents. (**a**) PARG-depleted U-2-OS cells were co-transfected with full-length GFP–EWS and the prion-like domains of EWS and FUS fused to RFP. Cells were laser microirradiated and time-lapse movie stills from the first 10 min after irradiation shown. Green arrows (upper panels) point to the recruitment of full-length GFP–EWS to DNA damage sites. Red arrows (middle panels) point to the redistribution of a prion-like domain-containing liquid droplet in the vicinity of the laser track. Note that the appearance of the distinct light-diffracting stripe at the laser microirradiated region is concomitant with the dissolution of the light-diffracting liquid droplet formed by the prion-like domains (lower panels). Prion domain containing droplets in distal regions of the nucleus and in the cytoplasm appeared stable during the period of observation. (**b**) PARG-depleted U-2-OS cells were transfected with full-length Tm-EWS. Cells were laser microirradiated and time-lapse movie stills from the first 10 min after irradiation shown. Red arrows (upper panels) point to the recruitment of Tm-EWS to DNA damage sites. Note that the full-length protein Tm-EWS dissolves into microdroplets at later time points. Scale bars, 10 μm.

We then asked whether the observed physicochemical changes are connected to the PAR-dependent assembly of LCD-containing proteins at sites of DNA damage. Indeed, and similar to abrogation of PAR formation, co-depletion of the three FET proteins by siRNA (see Supplementary Fig. 5j,k for knockdown efficiencies) abolished the early laser-induced changes in light diffraction (Fig. 4d; Supplementary Fig. 5l), and this affect was neither due to reduced amounts of DNA damage (Supplementary Fig. 5l,m) or PARP1/ARTD1 levels (Supplementary Fig. 5k) nor due to impaired DNA damage-induced PAR generation (Supplementary Fig. 5n). Conversely, ectopic overexpression of individual FET proteins greatly enhanced this cellular response, resulting in light-diffracting stripes even under mild laser conditions (Fig. 4e). Furthermore, we observed a rapid mobilization of prion-like domains in liquid droplets when liquid demixing was triggered in close vicinity by laser microirradiation (Fig. 5a), suggesting that these two compartments share physicochemical properties and can readily exchange constituents. In support of this notion, the transient assembly of full-length LCD-containing proteins at DNA damage sites frequently dissolved into microdroplets (Fig. 5b). Together, these data provide evidence that phase separation of LCD-containing proteins occurs at sites of DNA damage in a manner that is both dependent on local PAR formation and on the presence of PAR sensing, IDPs.

### Liquid demixing filters protein interactions

**Figure 6:**
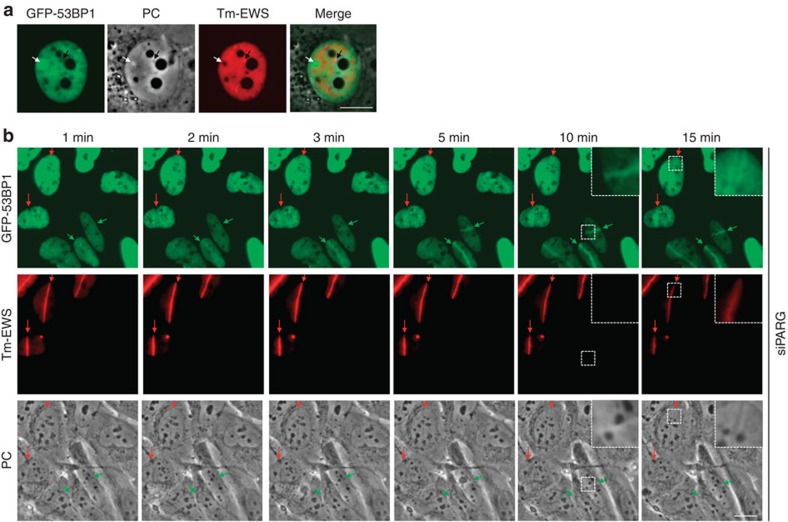
PAR-initiated liquid demixing can filter protein interactions at damaged chromatin. (**a**) Phase-contrast and fluorescent images depicting that sub-nuclear accumulation of the genome caretaker GFP–53BP1 correlates with decreased light diffraction and reduced levels of Tm-EWS (white arrows), while accumulation of Tm-EWS correlates with increased light diffraction and reduced levels of GFP–53BP1 (black arrows). (**b**) Movie snapshots from laser microirradiation experiments of GFP-53BP1/Tm-EWS co-expressing cells depicting reduced GFP–53BP1 accumulation at sites of EWS accumulation upon prolonged liquid demixing in PARG-depleted cells. Red arrows point at Tm-EWS accumulation, green arrows point at GFP–53BP1 accumulation. Scale bars, 10 μm.

The genome caretaker protein 53BP1 accumulates extensively on chromatin surrounding sites of spontaneous DNA lesions in proliferating cells^25^. We noticed that, unlike the PAR-dependent accumulation of LCD-containing proteins, 53BP1 accumulation did not cause an increase in light diffraction in phase-contrast images, but instead correlated with a marked decrease in light diffraction (Fig. 6a, white arrow). Moreover, the LCD-containing protein EWS was excluded from phase-contrast light 53BP1-decorated chromatin and, vice versa, we found 53BP1 to be always excluded from phase-contrast dark areas of EWS accumulation (Fig. 6a, white and black arrows, respectively). These findings are consistent with the observed exclusion of 53BP1 from FET prion domain-derived liquid droplets (Supplementary Fig. 4g), and suggest mutual exclusive occupancy of the sub-nuclear space by physicochemically distinct types of protein assemblies. To directly test whether the PAR-induced liquid demixing of IDPs at sites of DNA damage could affect the recruitment kinetics of globular genome caretakers, we expressed EWS in PARG-depleted cells (which show reduced degradation of PAR) and monitored simultaneously the accumulation of 53BP1, EWS and the accompanying changes in light diffraction on laser microirradiation. These experiments revealed that 53BP1 accumulation was delayed specifically in cells expressing EWS and was even prevented from binding to the areas with highest EWS accumulation (Fig. 6b). As observed before (Fig. 6a), EWS accumulation correlated with phase-contrast dark areas, while 53BP1 accumulation correlated with phase-contrast light areas (Fig. 6b, bottom panels). A quantitative analysis of 53BP1 foci induced by ionizing radiation confirmed that expression of EWS is able to suppress 53BP1 accumulation at damaged chromatin (Supplementary Fig. 6a). Interestingly, a more proximal component of the cellular response to DNA breakage, the large adaptor protein MDC1, was able to accumulate together with EWS at DNA damage-induced sites of PAR-initiated liquid demixing (Supplementary Fig. 6b,c). Of note, because we previously observed that in the nucleoplasm of otherwise unstressed cells both 53BP1 and MDC1 are excluded form prion-like liquid droplets (Supplementary Fig. 4f,g), these data indicate that the PAR-seeded liquid demixing at sites of DNA damage is a cellular means to differentially modulate the phosphorylation- and ubiquitylation-dependent assemblies of genome caretakers (MDC1 and 53BP1, respectively. See Discussion). Collectively, our data show that the PAR-seeded phase separation of IDPs is distinct from other types of sub-nuclear protein accumulation, in particular from the assembly of the 53BP1 chromatin compartment, and has the potential to modulate access of genome caretakers to sites of DNA damage.

### Isolated PAR chains accelerate LCD aggregation *in vitro*

**Figure 7:**
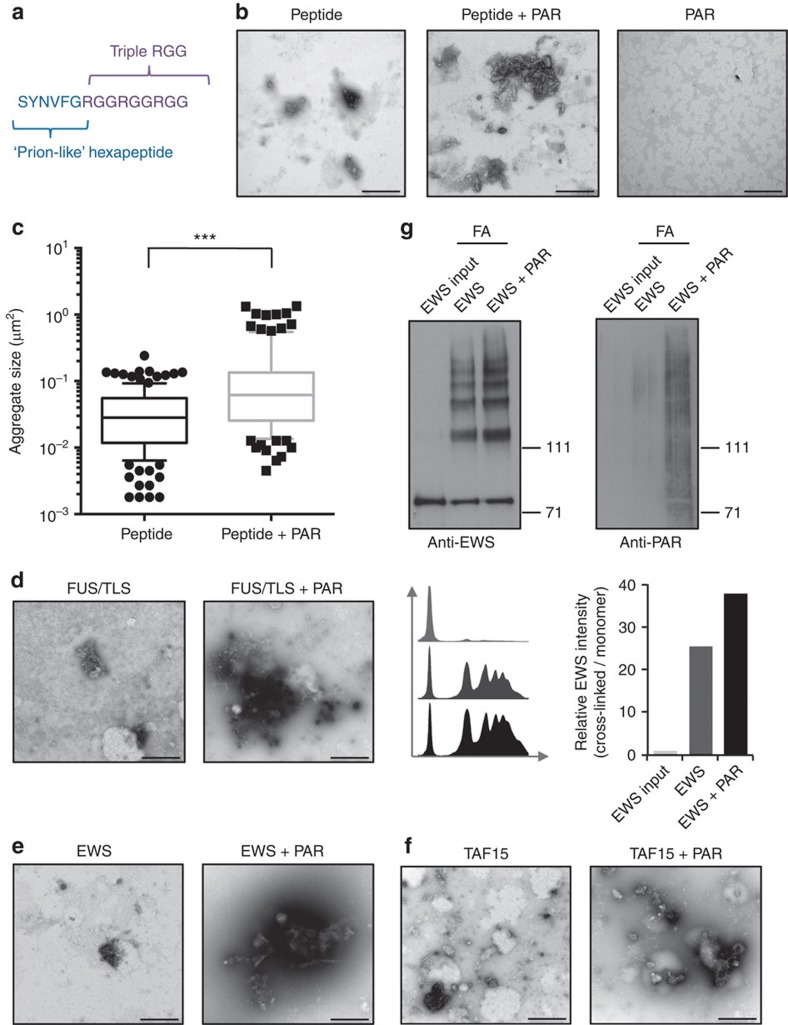
Isolated PAR chains accelerate LCD aggregation in a cell-free system. (**a**) Model peptide sequence designed to analyse PAR-seeded aggregation *in vitro*. The model peptide comprises a prion-like hexapeptide sequence followed by the three consecutive RGG repeats. (**b**) The model peptide was incubated at 37 °C for 24 h with or without sub-stoichiometric amounts of isolated, polydispersed PAR chains and spontaneous aggregates were analysed by transmission electron microscopy (TEM). (**c**) As in **b**, the model peptide was incubated with or without PAR, and aggregate sizes were determined from TEM images (*n*=137 for the peptide sample; *n*=116 for the peptide+PAR sample). ***P<0.0001 (Mann–Whitney test). (**d**) Full-length recombinant FUS was incubated at 37 °C for 24 h with or without sub-stoichiometric amounts of purified PAR and protein aggregates were analysed by TEM. (**e**) Full-length recombinant EWS was incubated at 37 °C for 24 h with or without sub-stoichiometric amounts of purified PAR and protein aggregates were analysed by TEM. (**f**) Full-length recombinant TAF15 was incubated at 37 °C for 24 h with or without sub-stoichiometric amounts of purified PAR and protein aggregates were analysed by TEM. All TEM experiments were repeated at least three times, and representative images are shown. Additional images are provided as Supplementary Fig. 8. Scale bars, 500 nm. (**g**) Full-length recombinant EWS was incubated with or without purified PAR, cross-linked in 0.4% formaldehyde (FA) for 15 min, and analysed by SDS–polyacrylamide gel electrophoresis (3–8% Tris–acetate). After detection of EWS complexes (left panel), the membrane was stripped and reprobed with an antibody against PAR (right panel). Signals from the anti-EWS western blot were quantified by ImageJ.

Since our data provide evidence for PAR-seeded phase separation of IDPs *in vivo*, an important prediction is that isolated PAR chains should enhance the intrinsic aggregation propensity of purified LCD-containing proteins by nucleating their assembly and consequently promoting their aggregation—an end point of liquid–liquid phase separation—in a cell-free system^26^. To directly test this prediction, we first employed a model peptide, comprising a short previously characterized prion-like sequence^27^ combined with a triple RGG repeat (Fig. 7a). As expected, this peptide spontaneously formed amorphous aggregates *in vitro* as revealed by transmission electron microscopy (TEM; Fig. 7b, left panel; Supplementary Fig. 7a, for additional examples of peptide aggregates). Strikingly, co-incubation with sub-stoichiometric amounts of purified PAR at physiologic pH conditions considerably enhanced the aggregation process, resulting in larger, more condensed aggregates (Fig. 7b, middle panel; Supplementary Fig. 7b, for additional examples of peptide aggregates). Comparable structures were absent in PAR alone or buffer control samples (Fig. 7b, right panel; Supplementary Fig. 7c,d). TEM-based quantification of peptide aggregates confirmed that larger aggregates were significantly enriched in samples incubated with PAR (Fig. 7c). Importantly, this increase in aggregate size was completely lost when PAR was degraded by recombinant PARG before peptide addition (Supplementary Fig. 7e). Together, these data provide evidence that isolated PAR chains nucleate and thus promote aggregation of intrinsically disordered model peptides *in vitro*. Consistent with a role of RGG repeats as PAR sensor via electrostatic interactions, charge reversal by substitution of arginine to glutamate abolished the PAR-dependent enhancement of the aggregation process (Supplementary Fig. 7f). Similar results were obtained when the model peptide contained an extra negative charge provided by amino-terminal serine phosphorylation (Supplementary Fig. 7g). Since PAR promoted the aggregation process also when a PARP inhibitor was present throughout the entire reaction (Supplementary Fig. 7h), ruling out residual enzymatic PARP activity, these data confirm that the PAR-seeded aggregation relies on non-covalent electrostatic interactions with PAR.

The model peptide employed in our TEM experiments combined a previously characterized aggregation-prone hexapeptide sequence^27^ with three PAR-responsive RGG repeats, thus mirroring the general build-up of the FET proteins. To directly test whether PAR would nucleate the aggregation also of the full-length proteins, we incubated recombinant FUS, EWS and TAF15 with sub-stoichiometric amounts of PAR. Similar to the model peptide, the full-length FET proteins formed spontaneous aggregates *in vitro* and these aggregates were consistently larger in the presence of PAR (Fig. 7d–f). As an independent approach to assess the PAR-mediated formation of higher-order protein structures, we performed formaldehyde cross-linking experiments with recombinant FUS and EWS in the absence and presence of PAR. Similar to the results of our TEM analyses, addition of purified PAR enhanced the formation of cross-linked protein dimers and multimers relative to the monomers (Fig. 7g; Supplementary Fig. 7i,j). Collectively, and consistent with our *in vivo* analyses, these data provide evidence for the intrinsic ability of PAR chains to nucleate aggregation of LCD-containing, IDPs.

## Discussion

**Figure 8:**
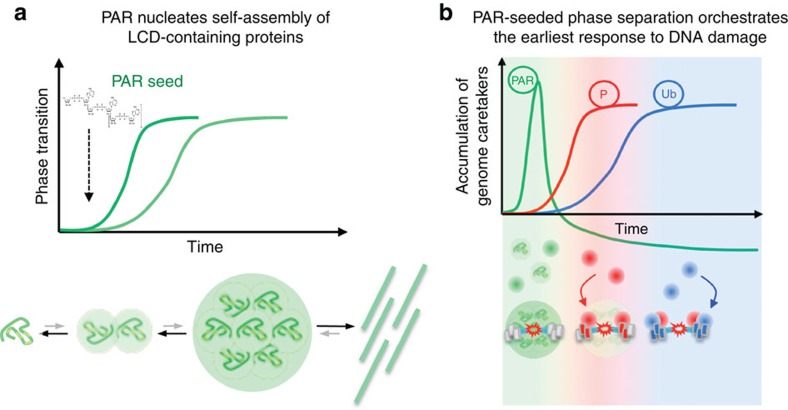
Model for PAR-nucleated liquid demixing of LCD-containing proteins. (**a**) Intrinsically disordered LCD-containing proteins undergo spontaneous self-assembly to generate higher-order structures. The initial kinetics during the nucleation phase of this process is relatively slow due to fast reverse reaction rates. However, molecular seeds can significantly accelerate the nucleation process, thereby help to overcome the kinetic barrier and drive the formation of higher-order structures; in case of excessive or lasting stimuli pathological fibrils or irreversible protein aggregates may form. We propose that the low complexity anionic biopolymer poly(ADP-ribose) (PAR) constitutes a molecular seed for the self-assembly of LCD-containing proteins. By virtue of its non-rigid structure and polydispersed nature, PAR can trap intrinsically disordered LCD-containing proteins and facilitate their dynamic assembly into higher-order structures. Under physiological conditions, the PAR-seeded assembly of LCD-containing proteins thus represents a liquid–liquid phase separation, with the potential to dynamically compartmentalize the subcellular space in a context-dependent manner. Under pathological conditions, derailed phase transitions may lead to the formation of less dynamic protein aggregates. (**b**) In the physiological context of the cellular response to DNA damage, PAR levels spike locally due to hyperactivation of PARP enzymes directly at DNA break sites, resulting in the rapid accumulation of various LCD-containing proteins. Accordingly, the greatly increased local concentration of LCD-containing proteins results in rapid phase separation and liquid demixing, providing cells with an opportunity to filter molecular interactions occurring on damaged chromatin. Dissolution of PAR-seeded liquid compartments paves the way for dedicated high-affinity key–lock interactions to unfold on the lesion-flanking chromatin allowing for the accumulation of genome caretakers such as 53BP1.

Using DNA damage as an archetypical biological setting in which PARP enzymes are rapidly activated in a spatially confined manner, our study introduces polynucleotide, nucleic acid-like, but in terms of sequence information non-coding PAR as seed for dynamic organization of the soluble nuclear space into distinct compartments with hallmarks of membrane-less organelles (Fig. 8a). Our experimental evidence is congruent with a recent theoretical conjecture about the emerging roles of PAR as organizer of cellular architecture and potential seed for protein self-assembly^28^, as well as with previous work on LCD-driven compartmentalization^29^ and liquid demixing by phase separation^30^,^31^. Moreover, our data suggest that local PAR formation may act as molecular sponge to outcompete interactions between LCD-containing proteins and their cognate polynucleotide RNA or DNA targets and promote rapid and context-specific reassembly in areas where PAR levels spike. Thus, local PAR formation can orchestrate intracellular phase transitions and their dimensions, allowing for their controlled formation and timely dissolution.

We find that the three FET proteins play a pivotal role in mediating the PAR-seeded formation of membrane-less organelles, but are probably just the tip of the iceberg of PAR-responsive, LCD-containing proteins. FUS, EWS and TAF15 were recently reported to belong to a metastable sub-proteome, which appears susceptible to aggregation due to high cellular concentrations relative to solubility, that is, due to ‘supersaturation'^32^. As supersaturated proteins, the FET members can be regarded as constantly poised for phase separation, and are thus ideally suited as prime responders to cellular events that nucleate liquid demixing. Although much remains to be learnt about the physiological functions of collective, multivalent low-affinity interactions of IDPs with PAR and the ensuing phase separation into liquid droplet-like environments, our data suggest that in response to DNA breakage such compartmentalization may help to coordinate the earliest stages of DNA repair via acting as transient interaction filter for genome caretakers (Fig. 8b).

An emerging theme in genome maintenance are inbuilt limitations governing chromatin responses to genotoxic stress, which likely evolved to prevent their excessive spreading or limit their duration^33^,^34^. Concentrating IDPs by PAR seeding bears the inherent risk of triggering their irreversible aggregation and raises the question of what are the means to control PAR-nucleated phase transitions. First, the transient nature of DNA damage-induced PARP activation ensures that the duration and extent of PAR formation is temporally constrained. Second, the steady-state activity of PARG results in rapid degradation of PAR and thereby prevents its excessive accumulation. However, given that, once initiated, the self-assembly of proteins into higher-order structures could become independent of the nucleating moiety, additional mechanisms must exist to prevent the irreversible protein accumulation. We envision that protein phosphorylation might be such an additional layer by providing extra negative charges to target molecules and thus reducing their affinity to the anionic polymer. Such modifications could prevent further protein interactions with PAR, as well as resolubilize proteins after PAR-initiated liquid demixing. Indeed, phosphorylation events were recently shown to control phase transitions in the contexts of stress granule dissolution, transcriptional activation and signalling^21^,^35^,^36^,^37^. In the context of the DDR, the kinases ATM, ATR and DNA-PK generate a pronounced protein phosphorylation cloud, which might help to ensure that PAR-nucleated protein assemblies remain transient. This scenario is consistent with our finding that the PAR-seeded compartment remains penetrable for the adaptor protein MDC1, which is required to amplify the phosphorylation signal on damaged chromatin. It would be also consistent with the formation of ‘anti-stripes', which we observed even under conditions of PARP inhibition, and which may coincide with the phosphorylation-dependent transcriptional repression in the vicinity of DNA damage^24^,^38^,^39^,^40^. Indeed, several hnRNPs and FET proteins are phosphorylated in response to DNA damage, often on multiple sites^14^,^38^, and the temporally shifted peaks of PAR-dependent and DDR kinase-dependent events (Fig. 8b) may thus underlie the biphasic nature of DNA damage-induced chromatin reorganization^41^. Of note, the same set of proteins associated with PAR-seeded liquid demixing are also covalently PARylated on genotoxic stress^42^, and an important question to address in the future is whether covalent modification of LCD-containing proteins with PAR assists phase separation, as proposed recently^28^, or, via a negative feedback mechanism based on electrostatic repulsion between PARylated protein sequences and chromatin-associated PAR, counteracts this process.

Cancer-associated translocations within the FET group of proteins, invariably leading to fusion proteins lacking the PAR sensing carboxyl-terminal RGG repeats, could reflect at least in part their impaired phase separation in response to local PAR formation, which may in turn undermine genome integrity. Indeed, Ewing sarcoma cells were recently shown to be exquisitely sensitive to PARP inhibitors^43^ and to a combination of PARP inhibition and radiation therapy^44^, and it is conceivable that under these conditions EWS dysfunction synergizes with impaired PAR seeding capacities, similar to what we found in colony formation assays. FUS was recently reported to be required for DNA repair by both non-homologous end-joining and homologous recombination^16^,^17^. Loss of FUS resulted in radiosensitivity^17^, chromosomal instability in mice^45^ and ALS patients with FUS mutations show increased DNA damage in the brain^16^. Less is known about the role of TAF15 for genome stability, but we observed that combined loss of all the three FET proteins leads to more severe accumulation of γH2AX in unchallenged conditions and higher levels of residual γH2AX after ionizing radiation than individual or pairwise depletion (data not shown), thereby suggesting that TAF15 also contributes to genome integrity maintenance.

Conversely to a situation of attenuated phase separation, under conditions when PAR generation is abnormally elevated, its timely degradation is impaired, or when mutations within supersaturated IDPs increase their aggregation propensity, dynamic phase transitions might turn into stable and irreversible protein aggregates associated with proteinopathies. Indeed, multiple mutations within the FET proteins and certain hnRNPs have been associated with diseases linked to pathological protein aggregation, most prominently with ALS and FTLD^2^,^13^,^18^. Along the same line, mice lacking PARP1/ARTD1 or treated with PARP inhibitors are protected from experimentally evoked parkinsonism^46^,^47^, whereas loss of PARG causes progressive neurodegeneration in *Drosophila melanogaster*^48^. Thus, PAR may influence protein homeostasis by regulating protein self-assembly for phase separation, linking PAR-seeded liquid demixing to pathological conditions characterized by abnormal protein aggregation. Our finding of a tightly controlled nucleation event for intracellular phase separation provides a conceptual framework to understand PAR-mediated protein assemblies as general regulatory principle of subcellular organization with implications for pathological protein aggregate depositions.

## Methods

### Statistical analysis

A total of 163 proteins associated with PAR^12^ were compared with unique protein identifiers of 148 proteins associated with RNA granules and 104 proteins precipitated by biotinylated isoxazole (b-isox)^5^, or to a control group of 225 annotated nuclear kinases. Right-tailed *P* values were obtained by Fisher's exact test, using a total of 20.203 UniProt-listed human proteins as reference (Fig. 1; Supplementary Data 2). Protein domain scores and ranks are according to Li *et al*.^2^, RG/RGG repeats according to Thandapani *et al*.^49^ (Supplementary Data 2). For comparing quantitative microscopy data and TEM images, *P* values were calculated by Mann–Whitney test (Fig. 7; Supplementary Figs 2 and 7). Unpaired *t*-test was used to calculate *P* values for colony formation (Supplementary Fig. 3).

### Cloning

Full-length human FUS (UniGene Hs.513522), EWS (UniGene Hs.374477) and TAF15 (UniGene Hs.402752) complementary DNA was amplified and inserted into pAcGFP-C1 and ptdTomato-C1 (both Clontech). Domain deletion constructs and constructs containing amino-acid substitutions were generated by standard PCR-based cloning.

### Cell culture and transfections

Human U-2-OS osteosarcoma cells (mycoplasma-free and authenticated by short tandem repeat (STR) analysis) were grown in DMEM containing 10% fetal bovine serum (Gibco). Plasmid transfections were performed with Lipofectamine LTX and Plus Reagent (Invitrogen) for 24 h; siRNA transfections were performed with Ambion Silencer Select siRNA using Lipofectamine RNAiMAX (Invitrogen) for 72 h.

### Laser microirradiation

Laser microirradiation experiments were performed on BrdU-pre-sensitized cells employing a custom-designed PALM MicroBeam equipped with a 355 nm ultraviolet-A pulsed laser (Zeiss)^20^.

### Light microscopy

Standard widefield, confocal, high-content microscopy and time-lapse imaging were performed essentially as described before^10^,^50^,^51^ and as detailed in the Supplementary Methods Section.

### Transmission electron microscopy

Lyophilized peptides (Biosyntan) were dissolved in 40 mM HEPES–KOH, 150 mM KCl, pH 7.4, for a final concentration of 0.5 mg ml^−1^, and sub-stoichiometric amounts of purified polydispersed PAR (Trevigen) were added (molar ratio of peptide:PAR (mono-ADP-ribose equivalents)=227:1). For experiments with full-length proteins, recombinant FUS (OriGene), EWS (OriGene) and TAF15 (Abnova) were used. Samples for TEM analysis were diluted in 40 mM HEPES–KOH, 150 mM KCl, pH 7.4 and sub-stoichiometric amounts of PAR were added (molar ratio FUS:PAR (mADPR equivalents)=53:1; EWS:PAR (mADPR equivalents)=41:1; TAF15:PAR (mADPR equivalents)=40:1). Where indicated, recombinant PARG (Adipogen) was incubated at a final concentration of 0.013 μg ml^−1^ for 6 h with purified PAR before addition of the peptide. All samples were agitated for 20–24 h at 37 °C and 1,200 r.p.m. (Eppendorf Thermomixer). TEM samples were then prepared by spreading the solution onto carbon-coated grids, followed by staining with 2% phosphotunastic acid, pH 7.4. Images were acquired on a Phillips CM 100 TEM. All experiments were repeated at least three times, and representative images are shown.

Further details and additional methods are provided in the Supplementary Methods Section.

## Additional information

**How to cite this article:** Altmeyer, M. *et al*. Liquid demixing of intrinsically disordered proteins is seeded by poly(ADP-ribose). *Nat. Commun.* 6:8088 doi: 10.1038/ncomms9088 (2015).

## Supplementary Material

Supplementary Information Supplementary Figures 1-7, Supplementary Methods and Supplementary References

Supplementary Data 1 PrePPI (https://bhapp.c2b2.columbia.edu/PrePPI/) search for putative SAFB1 interactions. Prediction likelihood ratios, database likelihood ratios and final probabilities are listed.

Supplementary Data 2 Cross-comparison of proteins associated with RNA stress granules, hydrogel-like structures, and PAR^11^,^12^. The first spreadsheet contains the complete protein lists, its overlap, prion scores according to Li et al.^13^, and RG/RGG repeats according to Thandapaniet al.^14^. Right-tailed p-values (Fisher's exact test) are provided in the second spreadsheet.

Supplementary Movie 1 Related to Figure 1, and demonstrating recruitment and exclusion kinetics of GFP-FUS to sites of laser micro-irradiations. The movie covers the first 15 minutes after laser micro-irradiation at 15 seconds intervals.

Supplementary Movie 2 Related to Figure 3, and demonstrating GFP-FUS 1-211 liquid droplets and their regular fusion and fission. The movie covers one hour at 2 minutes intervals.

Supplementary Movie 3 Related to Figure 4, and demonstrating persistent increase of light diffraction at the microlaser-generated sites of DNA damage in PARG-depleted cells. The movie covers the first 15 minutes after laser micro-irradiation at 15 seconds intervals.
