# Supplementary material for: Liquid demixing of intrinsically disordered proteins is seeded by poly(ADP-ribose)
Source: Nat Commun. 2015 Aug 19;6:8088. doi: 10.1038/ncomms9088 (PMC4560800; doi:10.1038/ncomms9088)
Supplement: Supplementary Information — Supplementary Figures 1-7, Supplementary Methods and Supplementary References [file ncomms9088-s1.pdf]

Supplementary Figures

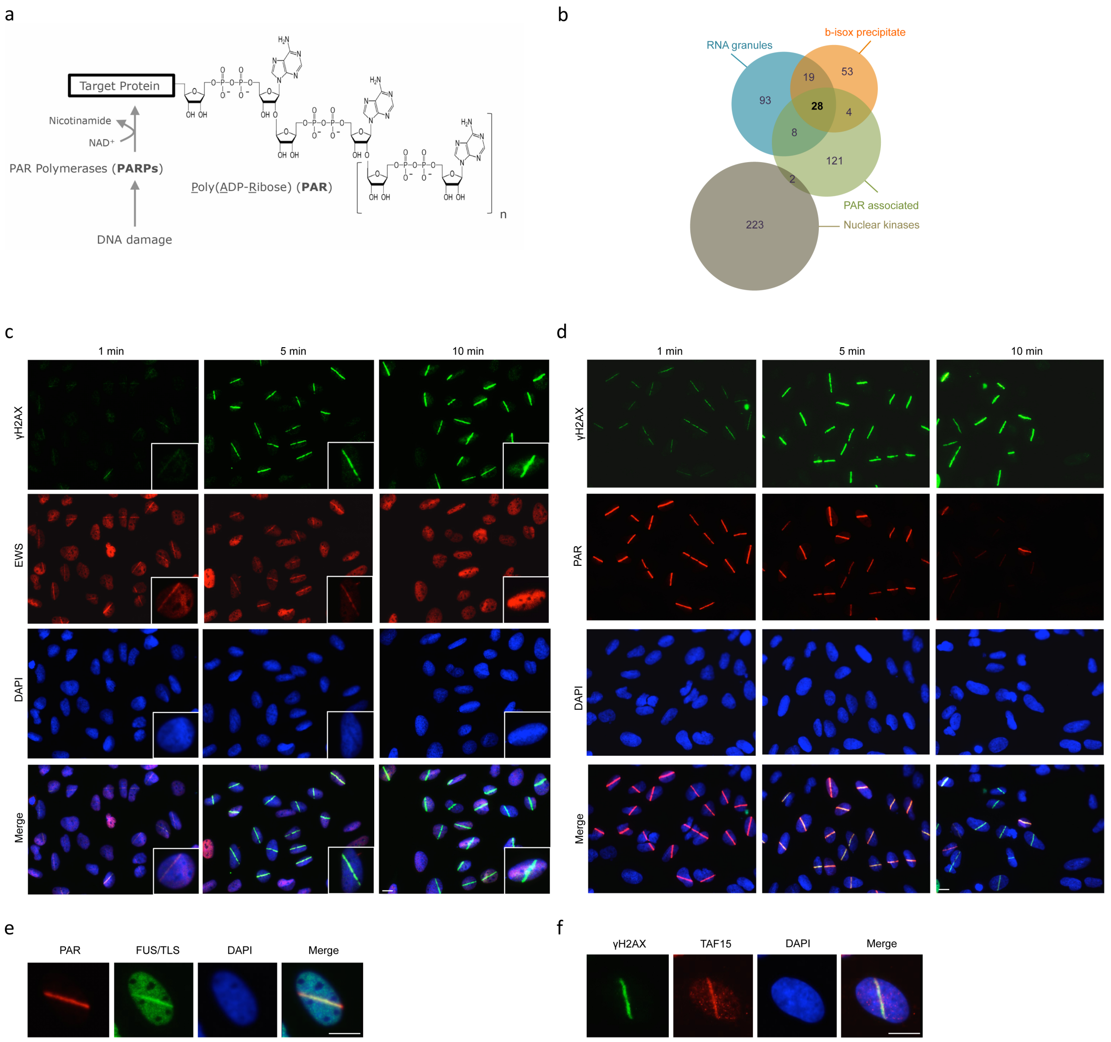

Supplementary Figure 1: Liquid demixing and PAR association is a common feature of LCD-containing proteins and endogenous LCD-containing proteins accumulate at sites of DNA damage with similar kinetics as PAR formation. (a) Chemical structure of poly(ADP-ribose) (PAR). PARP enzymes use NAD^+ as substrate to generate nicotinamide and chains of covalently linked ADP-ribose moieties. Each unit carries two negative charges, making the polymer highly anionic. PAR may contain several branching points (not shown) and can be covalently attached to several acceptor amino acids (mostly glutamates, aspartates, lysines, and arginines) in target proteins. **(b)** Combined overlap of proteins associated with RNA granules (blue), b-isox precipitates and *in vitro* generated hydrogels (orange), and PAR (green)[1,2]. A control group of 225 UniProt-annotated nuclear kinases is also shown. **(c)** Recruitment of endogenous EWS to sites of DNA breaks. U-2-OS cells were laser micro-irradiated, fixed after 1, 5, and 10 minutes, and stained for the DNA damage marker γH2AX and EWS. **(d)** Formation of PAR at sites of DNA breaks. U-2-OS cells were laser micro-irradiated, fixed after 1, 5, and 10 minutes, and stained for the DNA damage marker γH2AX and PAR. **(e)** Recruitment of endogenous FUS to sites of DNA breaks. U-2-OS cells were laser micro-irradiated, fixed after 5 minutes, and stained for PAR and FUS. **(f)** Recruitment of endogenous TAF15 to sites of DNA breaks. U-2-OS cells were laser micro-irradiated, fixed after 5 minutes, and stained for γH2AX and TAF15. Scale bars, 10 μm.

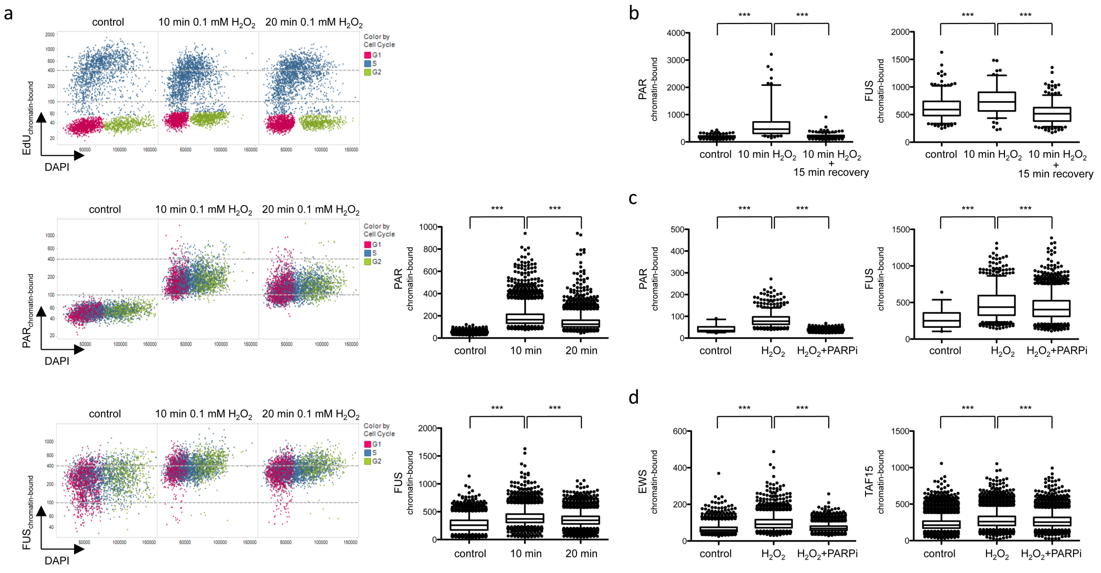

Supplementary Figure 2: PAR formation is associated with chromatin retention of LCD-containing proteins. (a) U-2-OS cells were labeled with EdU and treated as indicated with 0.1 mM H_2O_2. Cells were pre-extracted for 1 minute on ice in 0.2% Triton X-100, fixed and stained for PAR and FUS. Images of large cohorts of cells were taken by automated high-content microscopy. Mean EdU (Alexa488) intensities versus total DAPI intensities are depicted on the top. The color code corresponds to cell cycle phase. Below, mean PAR intensities (Alexa647) versus total DAPI intensities are depicted; the color code corresponds to cell cycle phase. A Whisker plot (5-95% percentile) of PAR intensities is shown on the right. At the bottom, mean FUS intensities (Alexa568) versus total DAPI intensities are depicted; the color code corresponds to cell cycle phase. A Whisker plot (5-95% percentile) of FUS intensities is shown on the right. **(b)** U-2-OS cells were treated with 0.1 mM H_2O_2 as indicated, pre-extracted, and stained for PAR (Alexa647) and FUS (Alexa488). Whisker plots (5-95% percentile) of PAR and FUS intensities are shown. **(c)** U-2-OS cells were treated for 10 minutes with 0.1 mM H_2O_2 with or without PARP inhibitor olaparib, pre-extracted, and stained for PAR (Alexa647) and FUS (Alexa488). Whisker plots (5-95% percentile) of PAR and FUS intensities are shown. **(d)** U-2-OS cells were treated for 10 minutes with 0.1 mM H_2O_2 with or without PARP inhibitor olaparib, pre-extracted, and stained for EWS (Alexa488) and TAF15 (Alexa647). Whisker plots (5-95% percentile) of EWS and TAF15 intensities are shown. *** $p < 0.0001$ (Mann-Whitneytest).

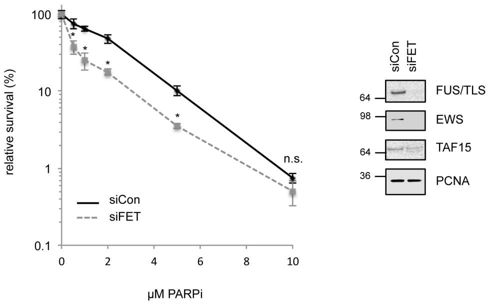

Supplementary Figure 3: FET proteins are effectors of PAR signaling. U-2-OS cells were transfected with control siRNA or siRNA targeting the three FET proteins. Clonogenic survival was measured as a function of increasing doses of the PARP inhibitor olaparib. P-values were calculated by unpaired t-test. * P-value < 0.05. A western blot to control for the knockdown efficiency of the combined siRNA treatment is shown on the right.

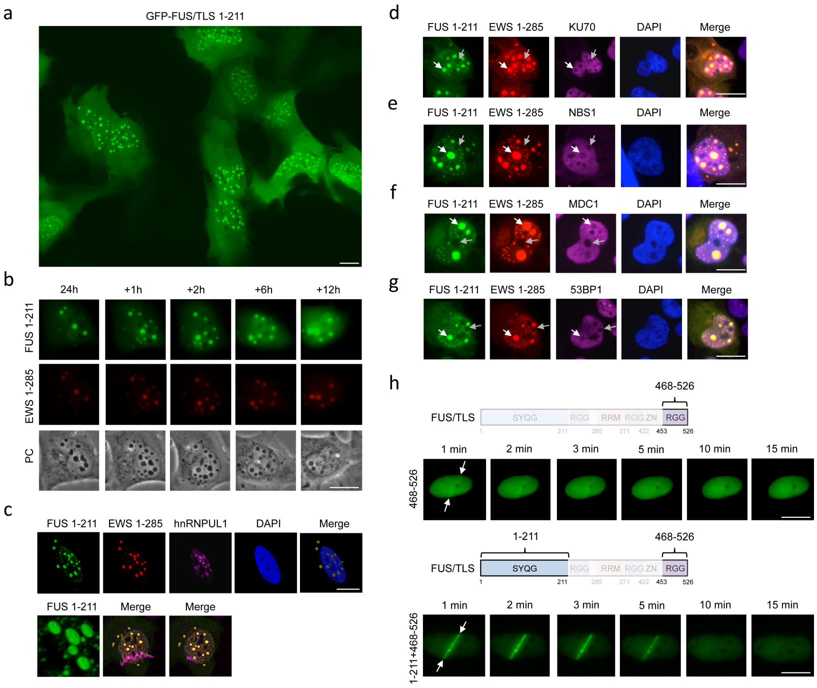

Supplementary Figure 4: Prion-like domains of LCD-containing proteins phaseseparate to form homo- and heterotypic droplets by liquid demixing. (a) GFP-FUS 1-211 was expressed for 24h in U-2-OS cells and spontaneous intracellular droplet formation was detected by fluorescence microscopy. **(b)** GFP-FUS 1-211 and Tomato-EWS 1-285 were co-expressed for 24h in U-2-OS cells and then monitored live by time-lapse imaging over a period of 12 hours in 15 minutes intervals. Movie stills are shown. PC, phase contrast. **(c)** 3D reconstruction of confocal z-stack images of the experiment shown in main Figure 3k, a higher magnification is shown below. Also shown are intensity plots demonstrating co-localization of hnRNPUL1 with liquid droplets inside, but not outside the cell nucleus, thus ruling out antibody cross-reactivity or bleed-through. **(d)** GFP-FUS 1-211 and Tomato-EWS 1-285 were co-expressed for 24h in U-2-OS cells to generate heterotypical liquid droplets. Cells were then fixed and stained for KU70. **(e)** Cells were treated as in (d) and stained for NBS1. **(f)** Cells were treated as in (d) and stained for MDC1. **(g)** Cells were treated as in (d) and stained for 53BP1. White arrows point to heterotypical liquid droplets, highlighting one example per cell and genome caretaker exclusion therein; grey arrows point to nucleoli, highlighting one example per cell and genome caretaker exclusion therein. **(h)** Recruitment kinetics of GFP-FUS 468-526 and GFP-FUS 1-211+486-526 to sites of laser micro-irradiation. Time-lapse movie stills from the first 15 minutes after irradiation are shown. White arrows indicate the orientation of the laser line. Scale bars, 10 μm.

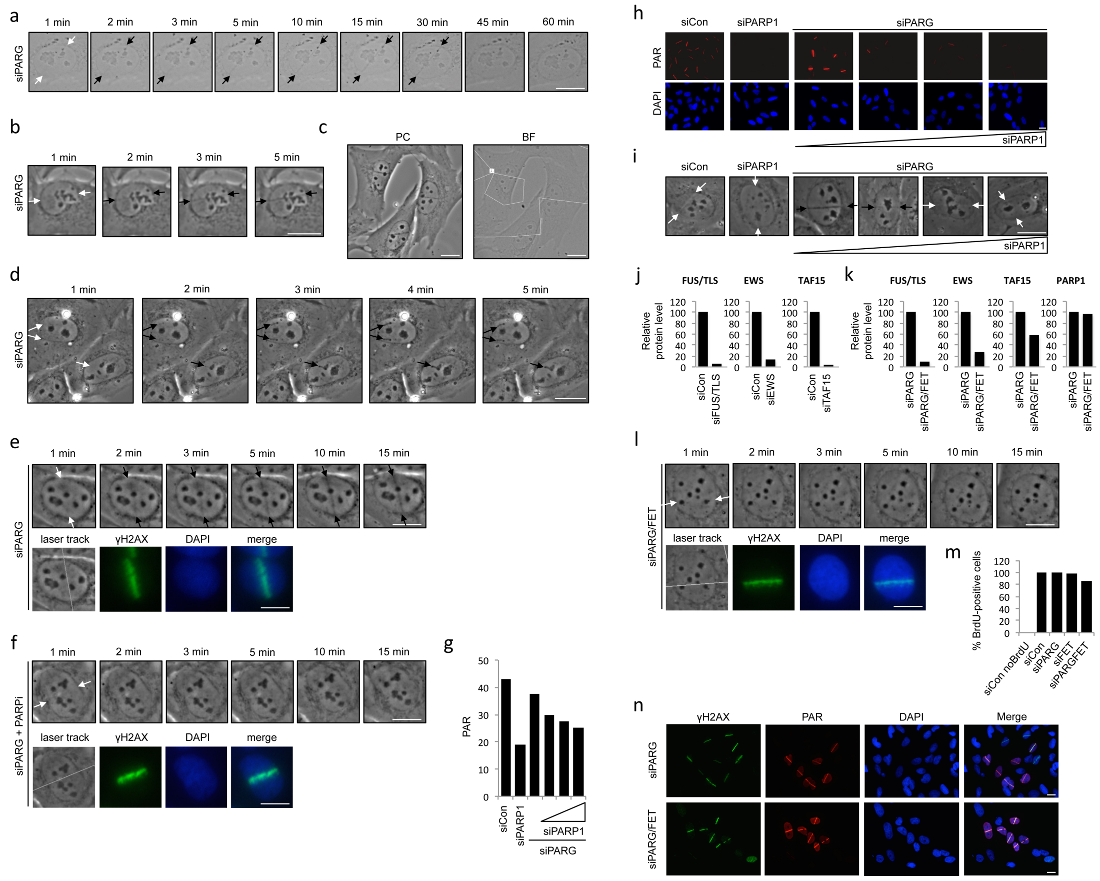

Supplementary Figure 5: PAR-dependent accumulation of LCD-containing proteins seeds liquid demixing at sites of DNA damage. (a) Bright-field images depicting the enhanced and prolonged, yet reversible generation of distinct light diffracting stripes at sites of laser micro-irradiation in PARG-depleted cells. **(b)** Phase contrast images depicting the early development of light diffracting stripes in PARG-depleted cells. **(c)** Phase contrast image depicting light diffracting stripes in the nuclei of laser micro-irradiated cells, but not in the surrounding cytoplasm. A bright-field image of the laser track is shown to the right. **(d)** Phase contrast images depicting areas of increased light diffraction in nuclei, which had been damaged only in one or two small sub-nuclear areas rather than across the nuclear diameter. **(e)** Phase contrast images depicting the enhanced and prolonged generation of distinct light diffracting stripes at sites of laser micro-irradiation in PARG-depleted cells. Following time-lapse imaging for 15 minutes, cells were fixed and stained for the DNA damage marker γH2AX. **(f)** Phase contrast images depicting the effect of PARP inhibition on the formation of distinct light diffracting stripes at sites of laser micro-irradiation in PARG-depleted cells. Following time-lapse imaging for 15 minutes, cells were fixed and stained for the DNA damage marker γH2AX. **(g)** Basal PAR levels in U-2-OS cells in response to PARP1 and PARG depletion as measured by high-content microscopy. Cells were transfected with the indicated combinations of siRNA; increasing amounts of PARP1 siRNA were used for final concentrations of 0.5 nM, 2.5 nM, and 12.5 nM. Cells were fixed and stained for PAR. Average mean nuclear PAR intensities of more than 1000 cells per condition are depicted. **(h)** As in (g), cells were transfected with the indicated siRNAs, laser micro-irradiated, fixed after 5 minutes, and stained for PAR and DAPI. **(i)** As in (g), cells were transfected with the indicated siRNAs and laser micro-irradiated. 5 minutes after DNA damage induction, phase contrast images were recorded. White arrows indicate the orientation of the laser line, black

arrows point to light diffracting dark stripes. Scale bars, 10 µm. **(j)** Knockdown efficiency of FUS, EWS and TAF15. Cells were transfected with the indicated siRNAs, fixed, stained with antibodies against the respective proteins, and analyzed by high-content microscopy. Normalized average mean nuclear fluorescence intensities of more than 1000 cells per condition are shown. **(k)** As in (j) cells were transfected with the indicated siRNA combinations and protein levels were measured by high-content microscopy using antibodies against the indicated proteins. Normalized average mean nuclear fluorescence intensities of more than 1000 cells per condition are shown. **(l)** Phase contrast images depicting the effect of FET knockdown on the formation of distinct light diffracting stripes at sites of laser micro-irradiation in PARG-depleted cells. Following time-lapse imaging for 15 minutes, cells were fixed and stained for the DNA damage marker γH2AX. **(m)** Cells were transfected with the indicated siRNAs for 48 hours, treated with BrdU for an additional 24 hours, and BrdU incorporation was analyzed by high-content microscopy of anti-BrdU stainings done under denaturing conditions. The percentage of BrdU-positive cells is depicted. More than 1000 cells were analyzed per condition. **(n)** Cells were transfected with the indicated siRNAs, laser micro-irradiated, fixed 5 minutes later and stained for γH2AX and PAR. Scale bars, 10 µm.

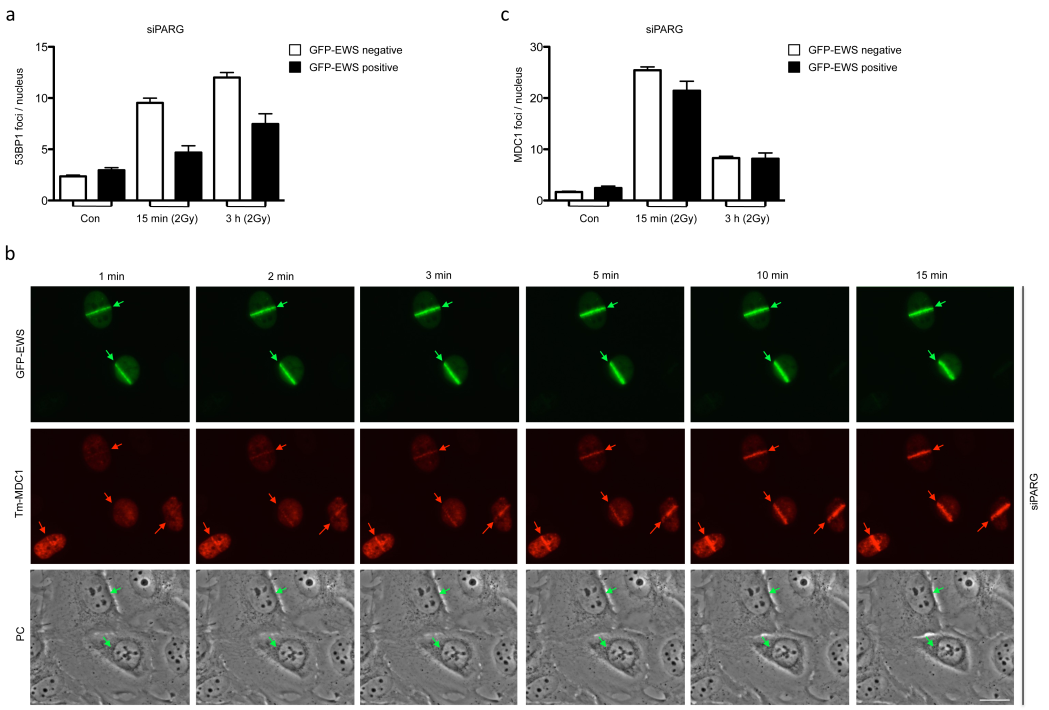

Supplementary Figure 6: PAR-initiated liquid demixing is permissive for MDC1 interaction with damaged chromatin. (a) PARG-depleted U-2-OS were transfected with GFP-EWS. Cells were irradiated with 2 Gy and fixed at the indicated time-points. Average 53BP1 foci counts per nucleus in GFP-negative and GFP-positive cells are depicted. Error bars: SEM. **(b)** PARG-depleted U-2-OS stably expressing Tm-MDC1 were transfected with GFP-EWS. Cells were laser micro-irradiated and time-lapse movie stills from the first 15 minutes after irradiation are shown. Green arrows (upper and lower panels) point to the recruitment of GFP-EWS to DNA damage sites. Red arrows (middle panels) point to the recruitment of Tm-MDC1 to DNA damage sites. **(c)** Average MDC1 foci counts per nucleus in GFP-negative and GFP-positive cells derived from the experiment shown in (a) are depicted. Error bars: SEM.

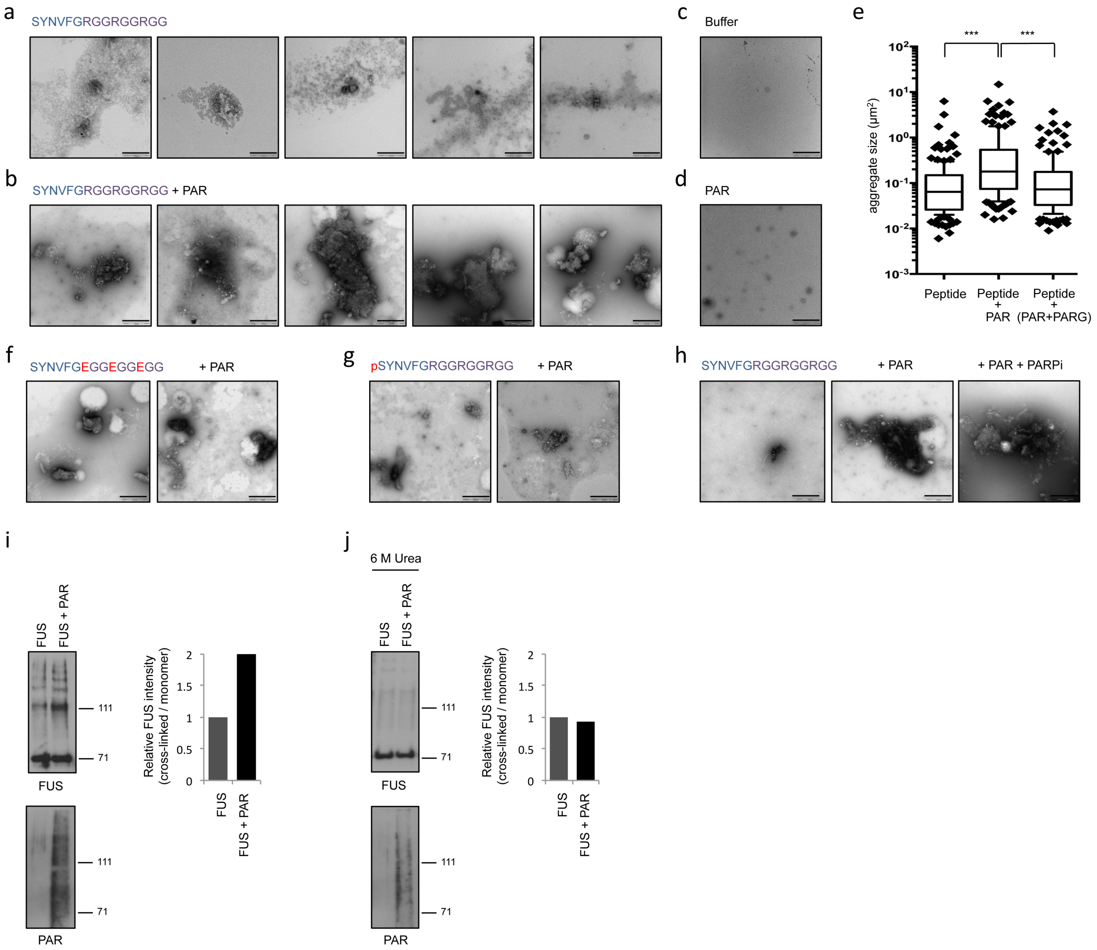

Supplementary Figure 7: Purified PAR chains promote LCD aggregation in a cell-free system. (a) Representative TEM images of the model peptide without PAR are shown. **(b)** Representative TEM images of the model peptide with PAR are shown. **(c)** TEM image of a buffer control sample. **(d)** TEM image of a PAR control sample. **(e)** The model peptide was incubated with or without PAR or PARG-treated PAR, and aggregate sizes were determined from TEM images (n=151 for the peptide sample; n=153 for the peptide + PAR sample; n=142 for the peptide + (PAR+PARG) sample). *** $p < 0.0001$ (Mann-Whitney test). **(f)** Representative TEM images of an R-to-E mutant peptide with and without PAR. **(g)** Representative TEM images of a phosphorylated peptide with and without PAR. **(h)** Representative TEM images of the model peptide with and without PAR and with PAR in the presence of 10 µM PARP inhibitor olaparib. Scale bars, 500 nm. **(i)** Full-length recombinant FUS was incubated with or without purified PAR, cross-linked in 0.4% formaldehyde (FA) for 15 minutes, and analyzed by SDS-PAGE (3-8% Tris-Acetate). After detection of FUS complexes, the membrane was stripped and re-probed with an antibody against PAR. **(j)** Reactions as in (i) were performed under denaturing conditions (6 M urea). Signals from anti-FUS western blots were quantified by ImageJ.

Supplementary Methods

Cloning

Full-length human FUS (UniGene Hs.513522), EWS (UniGene Hs.374477), and TAF15 (UniGene Hs.402752) cDNA was amplified by PCR and inserted into pAcGFP-C1 and ptdTomato-C1 (both Clontech). Amino-terminal deletion constructs to express the RGG-rich domains of the three FET proteins, FUS aa468-526, EWS aa445-656, and TAF15 aa320-592 were generated by standard PCR-based procedures using sequence-specific primers. The carboxyl-terminal deletion constructs to express the prion-like amino-termini of the three FET proteins, FUS aa1-211, EWS aa1-285, and TAF15 aa1-216 were generated by standard PCR-based procedures; in order to target the carboxyl-terminal deletion constructs to the nucleus, the non-classical PY-type nuclear localization signal, present at the carboxyl-terminus of the full-length wild-type FET proteins, was inserted just before the stop codon. Site-directed mutagenesis to replace multiple arginines present in the carboxyl-terminal FUS RGG repeats by serines was performed using the QuikChange Multi Site-Directed Mutagenesis Kit (Agilent Technologies) according to the manufacturer`s instructions. All constructs were verified by sequencing.

Cell culture and transfections

Human U-2-OS osteosarcoma cells (mycoplasma-free and authenticated by STR analysis) were grown in Dulbecco’s modified Eagle’s medium (DMEM) containing 10% fetal bovine serum (GIBCO). Plasmid transfections were performed with Lipofectamine LTX and Plus Reagent (Invitrogen) for 24 hours. siRNA transfections were performed with Ambion Silencer Select siRNA duplexes using Lipofectamine RNAiMAX (Invitrogen) for 72 hours. All siRNAs were purchased from Ambion/Life Technologies as Silencer Select Reagents and used at a final concentration of 25nM unless stated otherwise. When two or more siRNAs were combined for transfections, the total siRNA concentration was kept constant at 25nM. The following Silencer Select siRNAs were used in this study: siPARG (s16158), siPARP1 (s1098), siFUS (s5402), siEWS (s4886), siTAF15 (s15656). Negative control #1 from Ambion was used as a non-targeting control siRNA and is abbreviated as “siCon”.

Antibodies and drug treatments

The following antibodies were used in this study: rabbit polyclonal PAR antibody (Alx-210-890A-0100, Enzo Life Sciences, 1:1000), mouse monoclonal FUS antibody (sc-47711, Santa Cruz, 1:100), rabbit monoclonal TAF15 antibody (ab134916,

Abcam, 1:250), mouse monoclonal EWS antibody (DR1063, Merck Millipore, 1:1000), rabbit monoclonal hnRNPUL1 antibody (NBP1-40464, Novus Biologicals, 1:1000), mouse monoclonal γH2AX antibody (613401, Biolegend, 1:1000), rabbit polyclonal γH2AX antibody (ab2893, Abcam, 1:1000), mouse monoclonal KU70 antibody (ab2172, Abcam, 1:500), rabbit polyclonal NBS1 antibody (NB100-143, Novus Biologicals, 1:500), rabbit polyclonal MDC1 antibody (ab11171, Abcam, 1:2000), mouse monoclonal 53BP1 antibody (MAB3802, Merck Millipore, 1:1000), mouse monoclonal BrdU antibody (RPN202, GE Healthcare, 1:100). PARP inhibitor Olaparib (AZD228/Ku-0059436, Selleck Chemicals) was applied at a final concentration of 10 µM. For laser micro-irradiation and hydrogen peroxide experiments cells were exposed to Olaparib for 30 minutes prior to DNA damage induction. For *in vitro* analyses of PAR-nucleated peptide aggregation, the inhibitor was added prior to PAR addition and remained present throughout the reaction.

Colony formation

Colony formation assays were essentially performed as described[5]. Briefly, cells were transfected with siRNA, plated at low density, and treated with increasing doses of the PARP inhibitor olaparib. After 10–12 days, colonies were stained with 1% crystal violet in ethanol, and colonies with more than 50 cells were counted. All experiments were performed at least twice and in triplicates.

Laser micro-irradiation

Laser micro-irradiation experiments were performed as described previously on BrdU-pre-sensitized cells employing a custom-designed PALM MicroBeam equipped with a 355 nm UV-A pulsed laser (Zeiss) and using established protocols and laser energy conditions[6]. Accordingly, a laser output of 26-28 was used unless stated otherwise. In order to visualize early PAR-dependent changes in light diffraction by bright-field and phase contrast microscopy, the laser output was increased to 30-32 in siPARG cells, corresponding to an increase of about 50% (Figures 4b-d and Supplementary Figures 5a-f, i, and l) and to 34-36 in siCon cells, corresponding to an increase of about 100% (Figure 4a), as measured by a PD-300-3W photodiode power sensor (Ophir Photonics).

Time-lapse imaging

Time-lapse microscopy of laser micro-irradiated cells and of intracellular liquid droplet dynamics was performed under temperature-controlled conditions in CO_2-independent medium (GIBCO) containing 10% fetal bovine serum with LD Plan-

Neofluar 40x/0.6 Corr M27 and an LD Plan-Neofluar 40x/0.6 Corr Ph2 M27 dry objectives using AxioCam MRm, PALM RoboSoftware, and AxioVision Software. Image stacks were cropped using ImageJ to show single cells in high resolution, brightness and contrast were adjusted for better visualization; image stacks and movie frames are presented. Whenever single cells are depicted, representative cells were selected.

Immunostaining

U-2-OS cells were grown on 12 mm coverslips, fixed in 3% formaldehyde (Polysciences) in PBS for 12 minutes at room temperature, washed once in PBS, permeabilized for 5 minutes at room temperature in 0.2% Triton X-100 (Sigma-Aldrich) in PBS, and washed twice in PBS. Where indicated, cells were pre-extracted for 1 minute on ice in 0.2% Triton X-100 in PBS. All primary and secondary antibodies were diluted in filtered DMEM containing 10% FBS and 0.05% Sodium Azide. Primary antibody incubations were performed for 2 hours at room temperature. Coverslips were washed three times in PBS and then incubated with secondary antibodies (Alexa fluorophores, Life Technologies) for 1 hour at room temperature. Coverslips were washed once with PBS and incubated for 2 minutes with PBS containing 4',6-Diamidino-2-Phenylindole Dihydrochloride (DAPI, 0.5 µg/ml) at room temperature to stain DNA. Following three washing steps in PBS, coverslips were briefly washed with deionized water and mounted on 5µl Mowiol-based mounting media (Mowiol 4.88 (Calbiochem)/Glycerol/TRIS).

Standard fluorescence microscopy

Standard wide-field fluorescence microscopy was performed on a Zeiss Axio Imager A2 equipped with EC Plan-Neofluar 10x/0.3, 20x/0.5, 40x/0.75 dry objectives, and AxioCam ICc1 and AxioCam MRm cameras. To show single representative cells from replicated experiments, individual images were cropped, and brightness and contrast were adjusted for better visualization.

Confocal microscopy

Confocal microscopy was performed on a Zeiss LSM-780 confocal microscope with a 40x oil immersion objective Plan-Apochromat 403/1.3 (Figure 3k and Supplementary Figure 4c). Images were analyzed and 3D reconstruction of confocal z-stack images was performed using the Zeiss ZEN Microscope and Imaging Software (Supplementary Figure 4c).

High-content microscopy

Automated multichannel fluorescence microscopy was performed as described[7-9] on an Olympus ScanR system (motorized IX81 microscope) with ScanR Image Acquisition Software and UPLSAPO 10x/0.4, 20x/0.75 and 40x/0.9 dry objectives, fast-switching excitation and emission filter wheels for Dapi, FITC, Cy3 and Cy5 fluorescent dyes, an MT20 Illumination system, and a digital monochrome Hamamatsu C9100 electron multiplying (EM)-charge-coupled device (CCD) camera. For some experiments (Supplementary Figures 2a-d), automated multichannel fluorescence microscopy was performed on a Leica DMI 6000 inverted microscope equipped with a motorized stage, a fast Tri-band bandpass filter (DAPI, FITC, TX; excitation BP 387/11, BP 494/20, BP 575/20) and a Y5 filter (excitation BP 620/60, emission BP 700/75), a 12-bit monochrome EMCCD camera (Leica DFC 350 FX, 1392x1040 pixels, 6.4µm pixel size), a HC PLAN APO 10x (NA 0.4) air objective, and a HCX Plan APO 40x (NA 1.25-0.75) oil objective. Unbiased, automated image acquisition was performed with the Leica Matrix Screening Software. Image information of more than 1000 cells per condition, was acquired under non-saturating conditions, fluorescence intensities were quantified using the ScanR Image Analysis Software, and are depicted as relative arbitrary units (Supplementary Figures 2 and 5g, j, k, m). Scatter plots of cell populations were generated with Spotfire data visualization software (TIBCO) using raw data extracted from ScanR software (Figures 3d-f and Supplementary Figure 2a).

Transmission electron microscopy (TEM)

Lyophilized peptides were obtained from Biosyntan (Berlin, Germany). Peptide purity and molecular mass were confirmed by MALDI-TOF mass spectrometry. For TEM experiments, the peptides were dissolved in 40 mM HEPES-KOH, 150 mM KCl, pH 7.4, for a final concentration of 0.5 mg/ml (325 µM), as determined by absorption at 280 nm. Purified PAR was from Trevigen, USA. The following PAR concentrations are provided in mono-ADP-ribose (mADPR) equivalents, since the polymer preparation is polydispersed with PAR chain lengths ranging from 2 to 300 units: PAR was added to a final concentration of 1.45 µM (mADPR equivalents) to the peptides, resulting in a molar ratio of peptide:PAR (mADPR equivalents) = 227:1. For experiments with full-length proteins, recombinant FUS (OriGene, USA), EWS (OriGene, USA) and TAF15 (Abnova, Taiwan) were used. Purified FUS and EWS were supplied in 25 mM Tris-HCl, pH 7.4, 100 mM glycine, 10% glycerol; TAF15

was supplied in 50 mM Tris-HCl, 10 mM reduced Glutathione, pH 8.0. Samples for TEM analysis were prepared by diluting the protein stock solutions with 40 mM HEPES-KOH, 150 mM KCl, pH 7.4 to a final concentration 0.04 mg/ml (0.75 µM) FUS, 0.04 mg/ml (0.59 µM) EWS and 0.01 mg/ml (0.11 µM) TAF15. PAR was added to a final concentration of 14 nM to FUS and EWS (molar ratio FUS:PAR (mADPR equivalents) = 53:1; EWS:PAR (mADPR equivalents) = 41:1) and to a final concentration of 3nM to TAF15 (TAF15:PAR (mADPR equivalents) = 40:1). Where indicated, recombinant PARG (Adipogen) was incubated at a final concentration of 0.013 µg/ml for 6 hours with purified PAR prior to addition of the peptide. All samples were agitated for 20-24 hours at 37°C and 1200 rpm (Eppendorf Thermomixer) prior to TEM analysis. TEM samples were then prepared by spreading the solution onto carbon-coated grids, followed by staining with 2% phosphotunastic acid pH 7.4 for 1 minute. Images were acquired on a Phillips CM 100 transmission electron microscope equipped with a Veleta CCD camera and the iTIM acquisition software (Olympus). For quantification of aggregate size, the area of at least 100 aggregates per sample was quantified using ImageJ. Statistical significance was calculated based on the Mann-Whitney Test in Graphpad Prism Software. All experiments were repeated at least three times, and representative images are shown.

Formaldehyde cross-linking assays

Formaldehyde cross-linking reactions were adapted from[10]. 0.25 µg of recombinant protein were diluted in PBS pH 7.4 to a final concentration of 0.2 µM for FUS and 0.15 µM for EWS. PAR was added to a final concentration of 0.5 µM (mADPR equivalents). Samples were incubated for 15 minutes at 37°C, put to -20°C for 1h, incubated for 15 minutes at 37°C, and then cross-linked in 0.4% formaldehyde for 15 minutes at room temperature. Formaldehyde cross-linking was stopped by addition of glycine to a final concentration of 100 mM and boiling the samples in SDS-PAGE loading buffer for 5 minutes at 95°C. SDS-PAGE was performed with 3-8% Tris-Acetate gels. For denaturing conditions, the protein samples were prepared in 6 M urea.

Supplementary References

1 Kato, M. *et al.* Cell-free formation of RNA granules: low complexitysequence domains form dynamic fibers within hydrogels. *Cell* **149**, 753-767 (2012).

2 Isabelle, M., Gagne, J. P., Gallouzi, I. E. & Poirier, G. G. Quantitative proteomics and dynamic imaging reveal that G3BP-mediated stress granule assembly is poly(ADP-ribose)-dependent following exposure to MNNG- induced DNA alkylation. *Journal of cell science* **125**, 4555-4566 (2012).

3 Li, Y. R., King, O. D., Shorter, J. & Gitler, A. D. Stress granules as crucibles of ALS pathogenesis. *The Journal of cell biology* **201**, 361-372 (2013).

4 Thandapani, P., O'Connor, T. R., Bailey, T. L. & Richard, S. Defining the RGG/RG motif. *Molecular cell* **50**, 613-623 (2013).

5 Beli, P. *et al.* Proteomic investigations reveal a role for RNA processing factor THRAP3 in the DNA damage response. *Molecular cell* **46**, 212-225 (2012).

6 Bekker-Jensen, S. *et al.* Spatial organization of the mammalian genome surveillance machinery in response to DNA strand breaks. *The Journal of cell biology* **173**, 195-206 (2006).

7 Altmeyer, M. *et al.* The chromatin scaffold protein SAFB1 renders chromatin permissive for DNA damage signaling. *Molecular cell* **52**, 206-220 (2013).

8 Gudjonsson, T. *et al.* TRIP12 and UBR5 suppress spreading of chromatin ubiquitylation at damaged chromosomes. *Cell* **150**, 697-709, doi:10.1016/j.cell.2012.06.039 (2012).

9 Toledo, L. I. *et al.* ATR prohibits replication catastrophe by preventing global exhaustion of RPA. *Cell* **155**, 1088-1103, doi:10.1016/j.cell.2013.10.043 (2013).

10 Schwartz, J. C., Wang, X., Podell, E. R. & Cech, T. R. RNA seeds higher-order assembly of FUS protein. *Cell reports* **5**, 918-925, doi:10.1016/j.celrep.2013.11.017 (2013).

11 Kato, M. *et al.* Cell-free formation of RNA granules: low complexity sequence domains form dynamic fibers within hydrogels. *Cell* **149**, 753-767 (2012).

12 Isabelle, M., Gagne, J. P., Gallouzi, I. E. & Poirier, G. G. Quantitative proteomics and dynamic imaging reveal that G3BP-mediated stress granule assembly is poly(ADP-ribose)-dependent following exposure to MNNG-induced DNA alkylation. *Journal of cell science* **125**, 4555-4566 (2012).

13 Li, Y. R., King, O. D., Shorter, J. & Gitler, A. D. Stress granules as crucibles of ALS pathogenesis. *The Journal of cell biology* **201**, 361-372 (2013).

14 Thandapani, P., O'Connor, T. R., Bailey, T. L. & Richard, S. Defining the RGG/RG motif. *Molecular cell* **50**, 613-623 (2013).
